# Monolithic beam steering in a mid-infrared, surface-emitting, photonic integrated circuit

**DOI:** 10.1038/s41598-017-08916-9

**Published:** 2017-08-16

**Authors:** Steven Slivken, Donghai Wu, Manijeh Razeghi

**Affiliations:** Center for Quantum Devices, Department of Electrical Engineering and Computer Science, Northwestern University, Evanston, IL 60208 USA

## Abstract

The mid-infrared (2.5 < λ < 25 μm) spectral region is utilized for many purposes, such as chemical/biological sensing, free space communications, and illuminators/countermeasures. Compared to near-infrared optical systems, however, mid-infrared component technology is still rather crude, with isolated components exhibiting limited functionality. In this manuscript, we make a significant leap forward in mid-infrared technology by developing a platform which can combine functions of multiple mid-infrared optical elements, including an integrated light source. In a single device, we demonstrate wide wavelength tuning (240 nm) and beam steering (17.9 degrees) in the mid-infrared with a significantly reduced beam divergence (down to 0.5 degrees). The architecture is also set up to be manufacturable and testable on a wafer scale, requiring no cleaved facets or special mirror coating to function.

## Introduction

One of the hallmarks of modern optoelectronics is the ability to integrate multiple components onto a single piece of semiconductor, typically silicon or indium phosphide^1, 2^. The photonic integrated circuit (PIC) is an evolution of electronic integrated circuits and allows lasers, modulators, detectors, and filters to be fabricated on a wafer scale. This process avoids the need for external optical components and painstaking alignment, leading to low-cost, compact, robust and energy-efficient complex optical systems. Near-infrared PIC technology was initially driven by high bandwidth communications applications which led to the development of advanced growth and fabrication techniques^3^. PIC technology has now evolved to the point where it is now being customized for specific functionality.

A recent development which has received considerable attention is the use of PIC technology for all-electronic beam steering^4, 5^. Optomechanical systems used for steering beams (such as gimbal mounts) tend to be bulky, with limited speed, and are sensitive to temperature changes and mechanical shocks. Steerable PIC^-^based systems (ST-PIC), on the other hand, utilize wavelength, phase, or a reconfigurable resonator to shape and steer the beam, which can be done very quickly, without any moving parts. Applications for beam steering include free space communications, LIDAR/spatial mapping, imaging, and illumination.

PIC technology is also being customized for expanded spectral coverage. This includes the development of integrated optical technologies for use in the mid-infrared. A key component for realization of mid-infrared PICs has been the development of intersubband emitters based on semiconductor superlattices, which were first conceived of in the 1970s^6^. Intersubband emitter technology, in the form of the quantum cascade laser (QCL), has now achieved both high efficiency (53%) and high continuous wave output power (>5 W at room temperature) for discrete devices^7, 8^. The next technological step is to integrate this flexible mid-infrared emitter with other passive and active components. Already there have been some preliminary demonstrations of on-chip multiplexing for broadband spectral coverage and lab-on-a-chip functionality^9, 10^.

In this paper, we demonstrate a mid-infrared, steerable PIC (MIST-PIC) for directed energy applications. Wavelength agility, beam divergence, and beam steering are all managed with a combination of active and passive components. The resonator is completely self-contained, with light emitted from the surface. As a result, no cleaving is required, and inspection and testing of devices can be done on a wafer scale, which will significantly reduce production costs.

The concept starts with an electrically tunable sampled grating distributed feedback (SGDFB) QCL. This is a two section, active resonator, which controls the output wavelength via the optical Vernier effect^11^. The waveguide width in this region is similar to the free space wavelength of the device, which helps maintain beam quality. The SGDFB laser is combined with a grating outcoupler, which diffracts in-plane guided laser emission out of plane and reduces the beam divergence in one or two dimensions by transforming the size of the emitting aperture^12^. As the diffracted output angle of the grating is related to the input wavelength, beam steering is accomplished by tuning of the SGDFB laser.

## Results

### Linear grating outcoupler investigation

**Figure 1:**
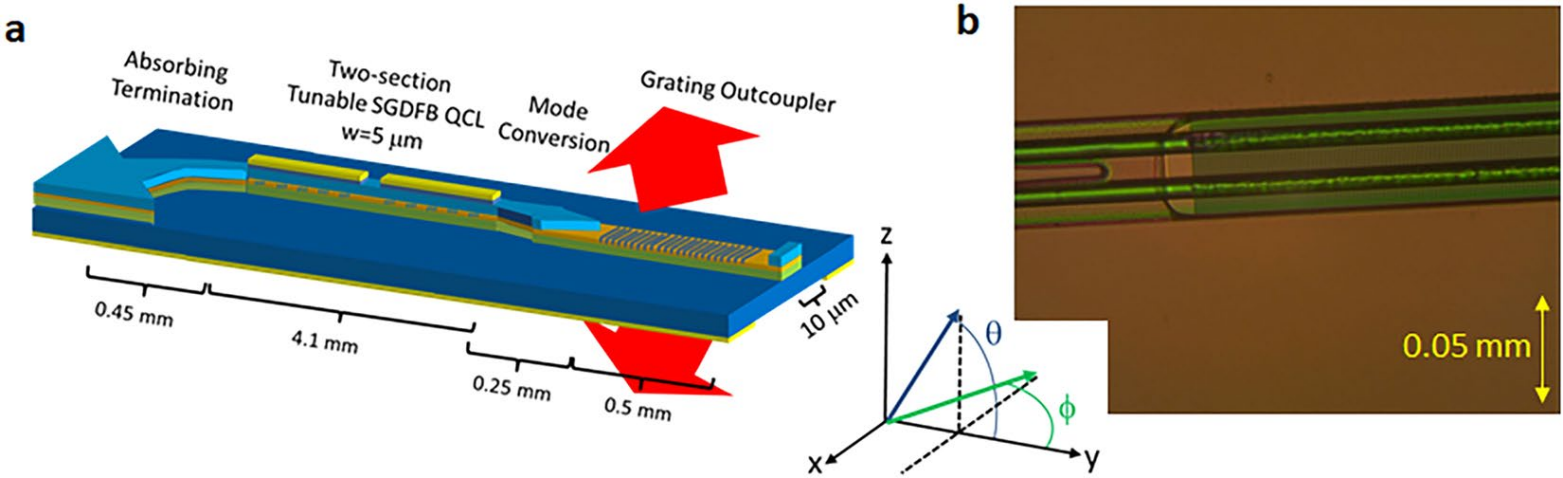
Monolithic mid-infrared beam steering architecture with linear grating. (**a)** Schematic of SGDFB laser integrated with linear grating outcoupler. (**b**) Optical microscope image of region connecting waveguide taper to linear grating outcoupler.

The first type of grating outcoupler explored is a linear grating which is integrated into a 10 μm wide passive waveguide (Fig. 1a). This design has a simple geometry which couples guided light into free space and transforms the beam divergence in the y-z plane (along θ). The grating outcoupler is connected to the SGDFB laser via a linear taper of the waveguide width from 5 to 10 μm (50 μm length), followed by a small spacer and a linear taper of the upper waveguide cladding width from 10 to 0 μm (180 μm length). The purpose of the taper is to transform the waveguide mode gradually in order to reduce reflections when light transitions between the active part of the device and the grating outcoupler region. In practice, the minimum taper width was 2 μm, limited by the fabrication method employed (see Methods). After the taper, the waveguide cladding is completely removed, which exposes the diffraction grating. The back section of the SGDFB laser emits into an absorbing termination, which is a passive 30-degree arc (w = 5 μm, r = 500 μm) connected to a linear taper whose width changes from 5 to 30 μm over a 200 μm length. While the schematic is shown as a ridge waveguide for clarity, the actual device was fabricated with a double channel waveguide. A top view of the fabricated cladding taper and grating region is shown in Fig. 1b.

**Figure 2:**
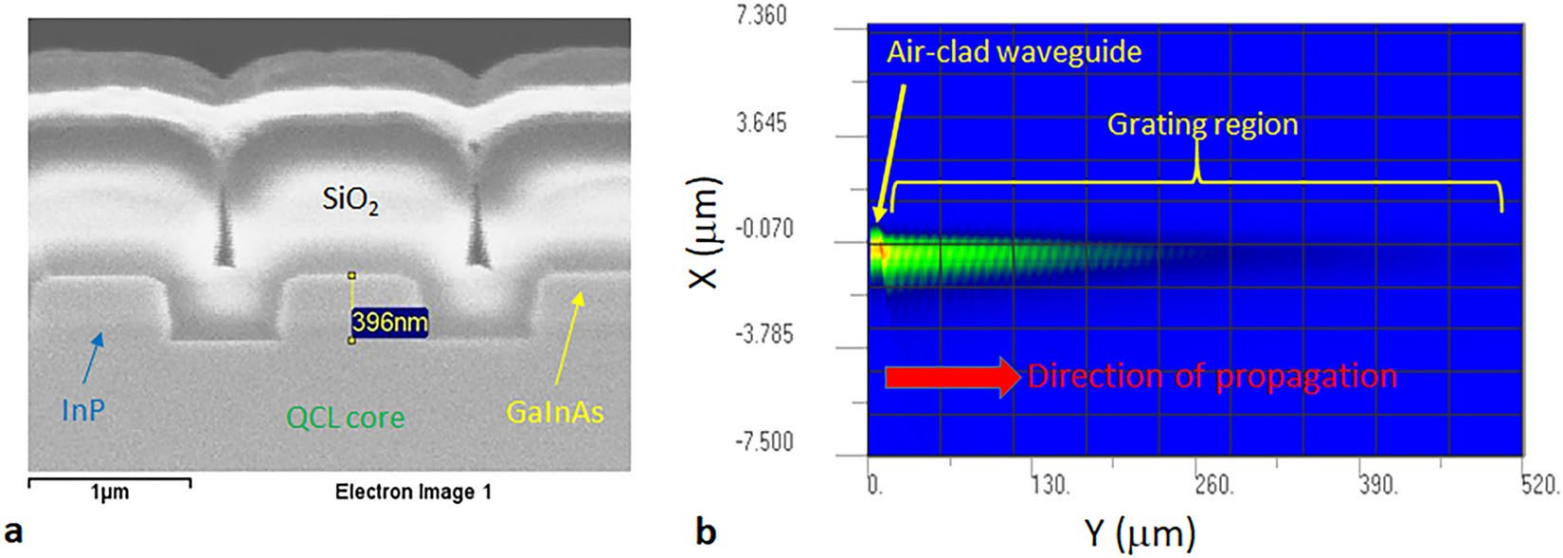
Grating geometry and simulation. (**a**) Cross-sectional scanning electron microscope image of grating outcoupler covered by SiO_2_. (**b**) 2D FDTD simulation of the Poynting vector in the y-direction for guided light propagating though a 500 μm long grating outcoupler.

The device waveguide structure is the same used in ref. 6, and is based on a 30 stage quantum cascade laser with a peak emission wavelength of 4.85 μm. A 300 nm thick GaInAs grating layer is positioned 100 nm above the QCL emitting region, for strong coupling of the grating to the waveguide’s fundamental transverse mode. For the SGDFB laser, the feedback grating is covered by InP, and has an estimated coupling coefficient (via the transfer matrix method) of >80 cm^−1^. For the grating outcoupler, the grating is covered by 1 μm of SiO_2_. A cross section of a grating outcoupler is shown in Fig. 2a. The depth is ~400 nm and the etched portion occupies ~40% of the grating period. A two-dimensional finite difference time domain (FDTD) simulation of light propagation inside the grating region for this geometry is shown in Fig. 2b. It is assumed that the input source is the fundamental transverse mode of the air-clad waveguide. According to the simulation, the diffraction loss coefficient, which is the attenuation coefficient for power diffracted out of the waveguide per unit length, is 41 cm^−1^. For a 500 μm long grating region, ~87% of the light is diffracted out of the waveguide.

**Figure 3:**
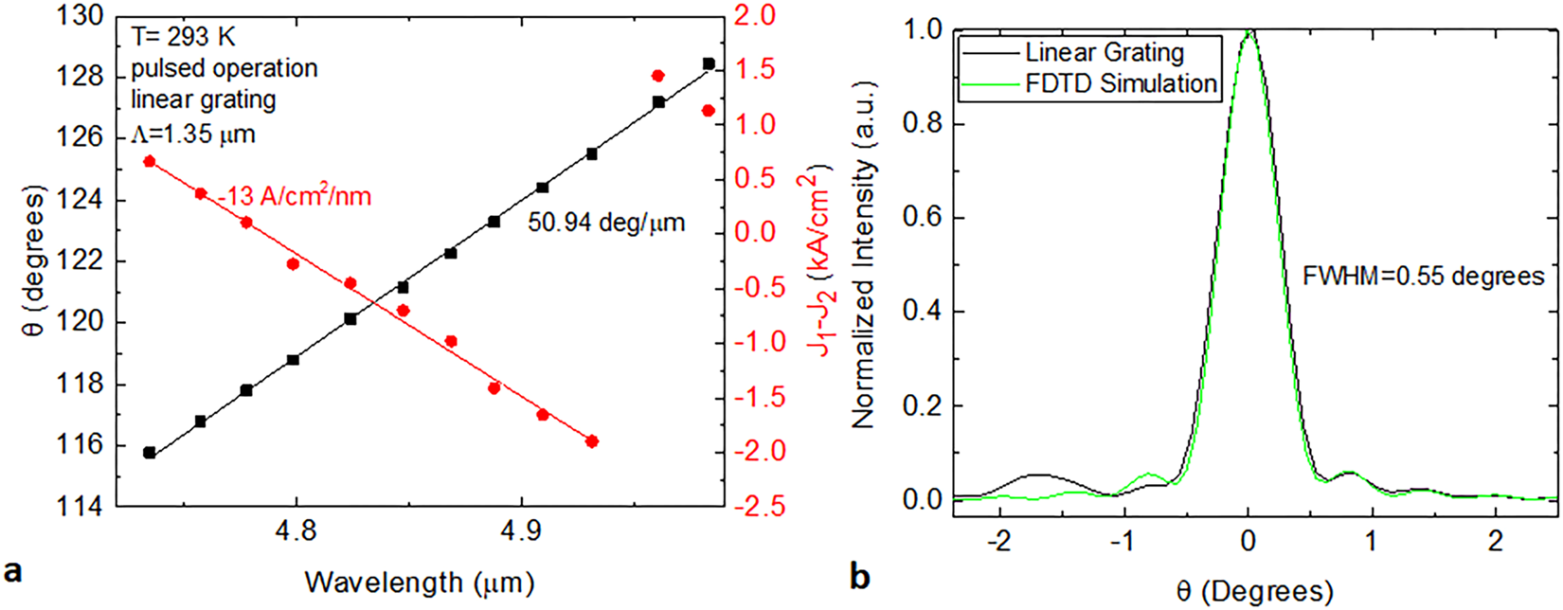
Tuning and steering behaviour for SGDFB lasers with linear grating outcouplers. (**a**) Steering angle and relative current density in the SGDFB sections as a function of emission wavelength. (**b**) Comparison of experimental and simulated far-field spectra in theta direction.

SGDFB lasers with linear grating outcouplers were tested in pulsed operation. Figure 3a shows the dependence of steering angle on wavelength for a typical device. The wavelength of the SGDFB laser is controlled by changing the relative current density, J_1_–J_2_, in the two sections of the device. The wavelength is adjustable over 248 nm with each nm of tuning requiring a 13 A/cm^2^ change in current density. For an outcoupler grating period, Λ, of 1.35 μm, the experimental steering rate is 50.94 degrees per micron of wavelength. Thanks to the wide wavelength tuning capability, this device shows a monolithic steering range of 12.6 degrees. This is significantly higher than a previous report of wavelength-based steering with an integrated source in the near-infrared, which exhibited <4 degrees of steering^13^.

The measured far-field along the theta axis matches well to simulation. Figure 3b overlays the measured far-field with a simulated curve based on the FDTD simulation in Fig. 2b. The θ axis of both curves has been offset to zero for ease of comparison. The lineshape and width are almost identical, with a full width at half maximum (FWHM) divergence of only 0.55 degrees. This is significantly smaller than edge emission from the SGDFB laser, which has a FWHM of 46 degrees. However, due to the narrow width of these lasers (10 μm), the lateral beam divergence FWHM is ~30 degrees.

### Tapered grating outcoupler investigation

**Figure 4:**
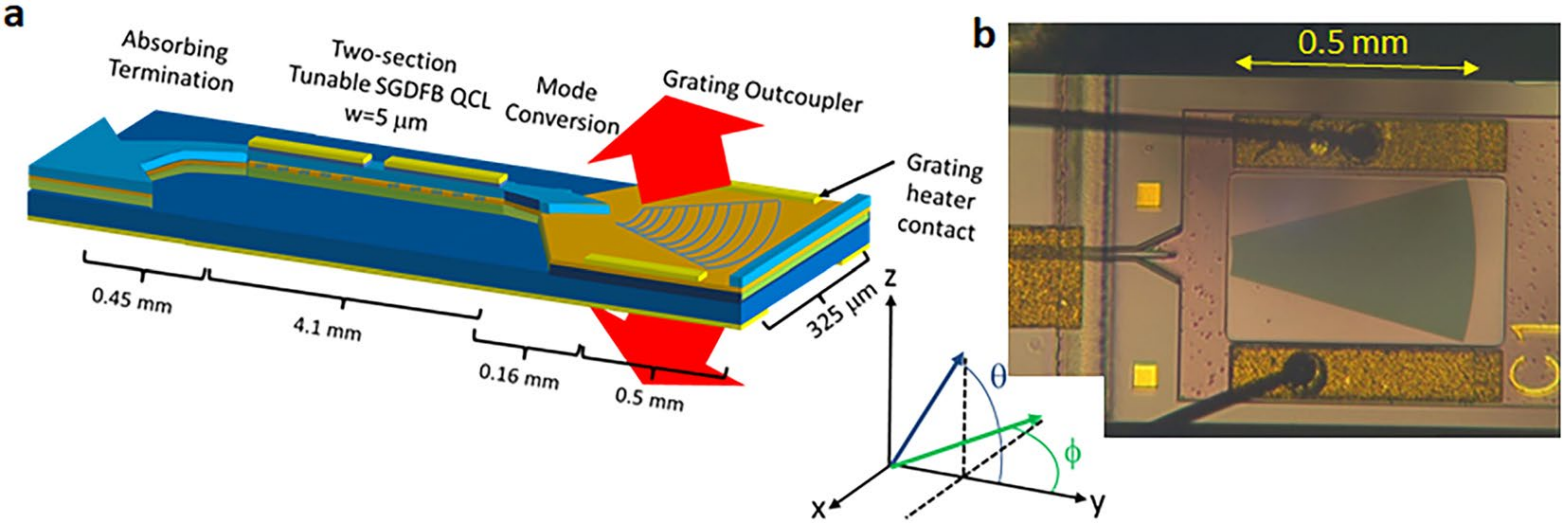
Monolithic mid-infrared beam steering architecture with tapered grating. (**a**) Schematic of SGDFB laser integrated with tapered grating outcoupler. (**b**) Optical microscope image waveguide taper, tapered grating outcoupler, and grating heater contract pads.

The second grating outcoupler explored is a tapered grating, shown in Fig. 4a. This type of grating is placed in an unbound region of the waveguide, where the input beam expands due to diffraction. The grating takes the form of circular arcs designed to match the phase front of the input beam. The benefit of this technique is that it provides a compact means to expand the output aperture laterally, which significantly reduces the output beam divergence. The arc origin should be placed near the end of the waveguide cladding taper, which in this case extends 90 μm from the end of the 10 μm waveguide. An additional feature of this architecture is the incorporation of a grating heater, which is simply two metal contacts on either side of the grating. This heater can be used to adjust the output angle for a given input beam wavelength. A top view of the fabricated cladding taper, grating region, and heater contacts is shown in Fig. 4b.

**Figure 5:**
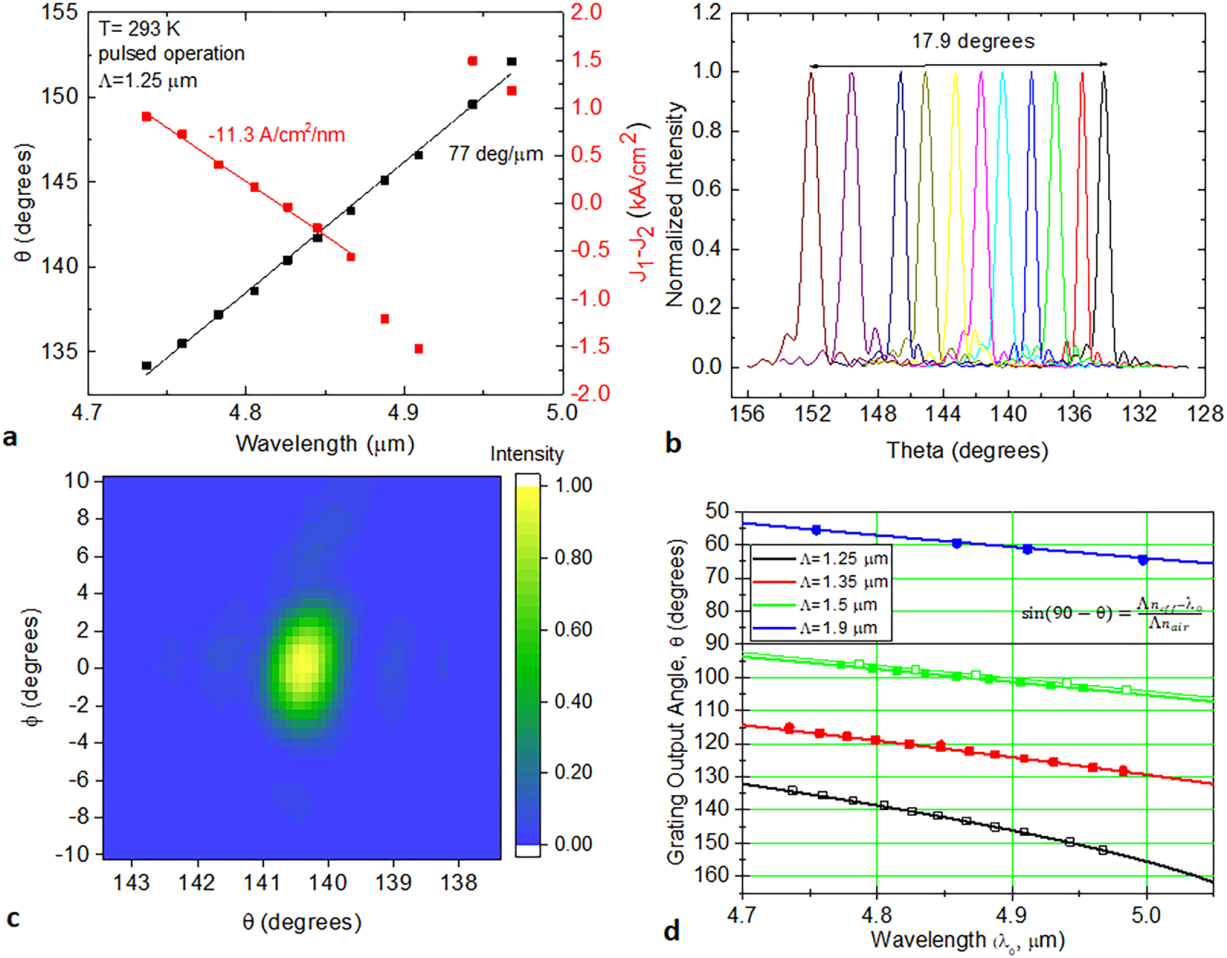
Tuning and steering behaviour for SGDFB lasers with tapered grating outcouplers. (**a**) Steering angle and relative current density in the SGDFB sections as a function of emission wavelength. (**b**) A collection of far-field scans along the theta axis (ϕ = 0) for different input wavelengths. (**c**) A two dimensional far-field scan of the tapered grating output beam at λ = 4.83 μm. (**d**) Comparison of experimental and calculated grating output angles for different input wavelengths and grating periods. Experimental data (symbols) and fit with solid lines following the formula given. Closed symbols = linear gratings. Open symbols = tapered gratings.

Testing results for a tapered grating with a period of 1.25 μm are shown in Fig. 5. Similar to the previous SGDFB laser, the wavelength tuning range is 240 nm. With the smaller grating period (Λ = 1.25 μm), however, the steering angular range is greatly expanded up to 17.9 degrees, with steering rate of 77 degrees per micron of wavelength. A collection of far-field scans along the theta axis (ϕ = 0) for different input wavelengths is shown in Fig. 5b.

A two dimensional far-field measurement was made for this device as well for an output wavelength of λ = 4.83 μm, which is shown in Fig. 5c. The spot is nearly symmetric, with a FWHM in the phi direction of ~5 degrees. The beam divergence demonstrated is significantly smaller than that from a 10 μm wide edge-emitter (~30 degrees), which indicates significant beam spreading in the grating region. The divergence can be reduced even more in the future by allowing the beam to expand further before entering the grating region. Even in the current configuration, however, the beam is significantly easier to collimate than a standard edge emitter.

In total, four different grating outcoupler periods (Λ = 1.25, 1.35, 1.5, and 1.9 μm) were tested in two different configurations (linear and tapered grating) in order to study the steering behaviour at different output angles. Figure 5d shows the comparison of the experimental steering angles with a theoretical fit based on simple diffraction theory. The only fit variable is n_eff_, which is the effective index of the propagating mode in the grating region. For all linear gratings, n_eff_ was fit to be 3.07; for tapered gratings, n_eff_ was fit to be 3.09. The highest slope region is observed in the region with the smallest lambda, which can be utilized to maximize the steering for a given wavelength tuning range.

**Figure 6:**
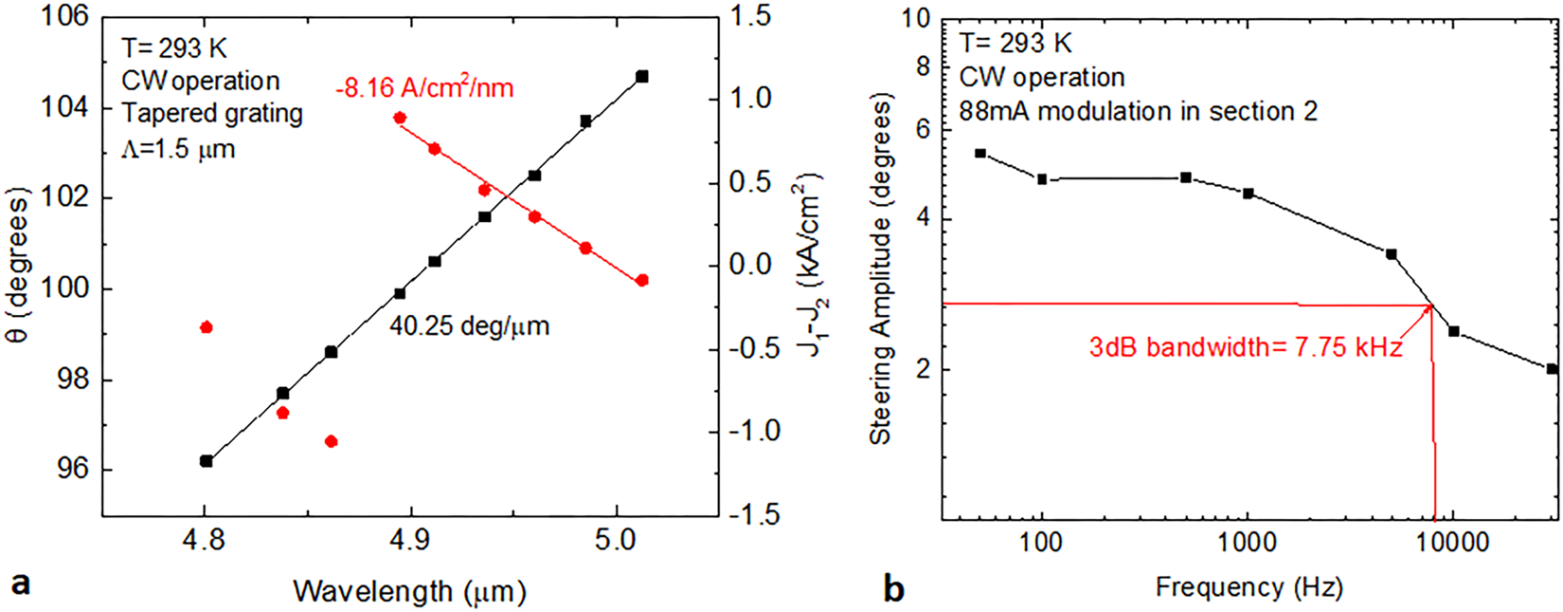
Tuning, steering, and modulation characteristics for SGDFB lasers with tapered grating outcouplers. (**a**) Steering angle and relative current density in the SGDFB sections as a function of emission wavelength. (**b**) Measured steering angle amplitude as a function of modulation frequency. Lines connecting data are a guide for the eye.

The SGDFB lasers with tapered grating outcouplers were also tested in CW operation. Figure 6a shows the dependence of steering angle on wavelength for a device with Λ = 1.5 μm. The wavelength is adjustable over 212 nm with each nm of tuning requiring an 8.16 A/cm^2^ change in current density. With this outcoupler grating period, the experimental steering rate is 40.25 degrees per micron of wavelength, which is nearly identical to a linear grating outcoupler with the same period. In CW operation, this device shows a monolithic steering range of 8.4 degrees.

For many illumination/countermeasure applications, the speed at which the beam can be steered is a critical factor for system performance. Though the full steering range typically requires two current inputs to be adjusted, a significant portion of this range can be accessed by modulating a single current input. With a constant current applied to SGDFB section 1 (the section farther from the grating outcoupler), a modulation of 88 mA in section 2 is capable of steering the beam by 5.2 degrees. With a stable bias above threshold, additional current was applied to section 2 in the form of a square wave with variable frequency. The steering amplitude was measured as a function of frequency and is plotted in Fig. 5b. Though the packaging has not yet been optimized for speed, the 3 dB bandwidth of the steering (and tuning) mechanism is shown to be ~7.75 kHz, which is sufficient for many imaging applications.

### Grating heater investigation

**Figure 7:**
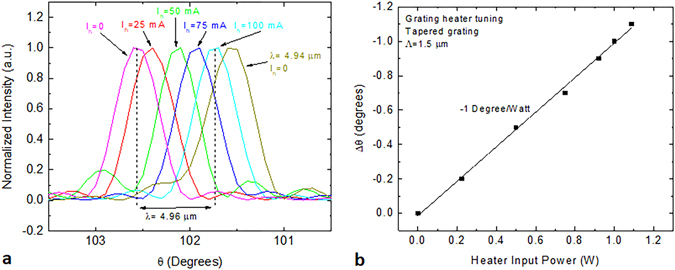
Grating heater tuning characteristics. (**a**) A collection of far-field scans along the theta axis (ϕ = 0) for different heater current, I_h_, values and input wavelengths. (**b**) Change in steering angle as a function of the input power to the grating heater.

Another feature built into the tapered grating device is a grating heater, which can be used to fine tune the steering angle. This is extremely important in situations where there is some dependence on atmospheric transmission with wavelength. A collection of far-field scans along the theta axis (ϕ = 0) for different heater current, I_h_, values is shown in Fig. 7a. At current values up to 100 mA, the heater is shown to bridge between two curves at different wavelengths which are approximately 1 degree (or 20 nm) apart. Taking the applied voltage into account, the heater power tuning curve is shown in Fig. 7b. A linear fit to the measured far-field spectra gives a slope of −1 degree per Watt of applied power. This should provide sufficient compensation to avoid high density water absorption peaks in the mid-infrared, which are typically less than 5 nm wide.

## Discussion

Though the beam steering demonstrated is already unprecedented for a wavelength-controlled ST-PIC with an integrated source, there is significant potential for increased tuning (and steering) with this QCL-based architecture. The gain bandwidth of these lasers is extremely wide, and a similar laser core was previously demonstrated with over twice the tuning range using an advanced SGDFB design^14^. In addition, by making use of heterogeneous QCL design principles, the output wavelength can be further adjustable over many microns^8, 15^.

Another potential improvement is the integration of a QCL-based optical amplifier. As demonstrated previously, the combination of a SGDFB source with an integrated amplifier can dramatically improve the output power. Edge emission of several Watts has already been demonstrated^16^.

In conclusion, the MIST-PIC platform, which allows for wide wavelength tuning, improved beam quality, and high speed beam steering, has been experimentally demonstrated. A local oscillator with a wavelength tuning range of 248 nm near a wavelength of 4.85 μm has been integrated with mode conversion structures in order to launch power into a passive region which contains a grating outcoupler. A steering range of up to 12.6 degrees has been demonstrated, with a beam divergence of 0.5 × 6 degrees. Beam steering with this mechanism is very fast, achieving a 3 dB bandwidth of 7.75 kHz for single current square wave modulation. Fine adjustment of output angle has also been realized with an additional grating heater, which can be used to maximize atmospheric transmission at specific angles. The overall architecture is modular, and represents the first step towards realizing additional MIST-PIC functionality such as 2-D beam steering and multi-Watt power output.

## Methods

### Growth and fabrication

The wafer growth started with a 1 μm n-doped (N_d_ = 2 × 10^16^ cm^−3^) InP buffer layer and a 30-stage QCL core designed for emission at λ = 4.85 μm. A 100 nm InP layer and a 300 nm lattice-matched GaInAs layer (both with N_d_ = 2 × 10^16^ cm^−3^) were grown on top to provide a closely coupled grating for the sampled grating device. These layers were grown using gas-source molecular beam epitaxy. Diffraction gratings were defined in the grating layer using e-beam lithography and plasma etching. Both SGDFB and outcoupler gratings are defined in a single exposure in order to minimize alignment errors.

The design and method of operation for the SGDFB laser is described in ref. 6. The sampled gratings in the SGDFB sections utilized a grating period of 785 nm with 18 grating periods per sampling period. Each section is made up of 12 repeated grating bursts, with a spacing of 170.9 and 158.8 μm for the first and second sections of the SGDFB laser, respectively. The grating outcouplers were defined as uniform linear and curved grating section with a length of 500 μm. and a grating period of 1.25, 1.35, 1.5, or 1.9 μm. After patterning, the top InP waveguide cladding and cap layers were grown using low pressure metalorganic chemical vapour deposition. The cladding layer is 3 μm thick with n-doping varied from 2 to 20 × 10^16^ cm^−3^. The cap layer is 1 μm thick with n-doping of 2 × 10^19^ cm^−3^.

Electrical isolation regions were next formed using photolithography and wet etching. These regions were defined in-between the SGDFB laser sections for independent bias control and at either end of the SGDFB laser to minimize leakage current leakage into the passive waveguide sections. The isolation channel used for the SGDFB laser is 100 um wide and 2 um deep, and it provides >1 kΩ of electrical isolation with minimal optical reflection.

Waveguide definition started with the regions around the grating outcouplers and includes the lateral waveguide cladding tapers used for mode conversion and power transfer. Plasma etching was used to etch away the cladding and cap layers down to the top of the grating in this region. After redefining the area around the gratings, an additional wet etching step was used to selectively remove the rest of the InP around and in-between grating features.

The SGDFB laser, absorbing termination, and mode expansion taper double channel waveguides were then defined, after careful alignment to the waveguide tapers, using photolithography. Plasma etching was used to etch channels through the epitaxial layers into the substrate. Following waveguide definition, a 1 μm thick layer of SiO_2_ was deposited as electrical insulation. For top contact formation, windows were opened on top of the SGDFB laser waveguide and on either side of the tapered grating outcouplers. Ti/Au was utilized as the top contact metal, and these regions were electroplated for a total metal thickness of 2.7 μm.

Following this, the substrate was thinned to 200 μm and polished. Bottom contact formation included the definition of emission windows aligned to the grating outcouplers in order to allow for direct substrate emission and minimize reflections that might cause distortion of the laser far-field pattern. The bottom contact metal consists of a multilayer of AuGe/Ni/Au.

### Device Testing

All devices were electrically checked prior to die singulation and packaging, which led to efficient packaging and excellent testing yield. Dies were bonded epilayer-up to copper submounts with indium solder. In order to have unobscured emission from the grating outcoupler, these regions were allowed to extend out beyond the copper submount. This incurs no penalty for the passive grating structures and actually allows for lower power operation of the grating heater.

For pulsed operation, the SGDFB sections were driven by two synchronized 100 ns pulses at a repetition rate of 100 kHz. Added dc bias was then applied to the two sections independently to achieve tuning of the laser output wavelength. For CW operation, two independent dc drivers were used to bias the SGDFB sections. A third dc driver was used to drive the grating heater, as necessary. Laser submounts were mounted on a temperature controlled-stage and held at a temperature of 293 K.

SGDFB laser output wavelength was measured using a vacuum FTIR (Bruker IFS 66v/S) with a HgCdTe photodetector and a resolution of 0.125 cm^−1^ to ensure single mode operation for steering measurements. Far fields patterns were obtained with a computer-controlled dual axis goniometer and a mercury cadmium telluride (MCT) detector.

For the steering modulation experiment, a low noise driver (Wavelength Electronics QCL2000LAB) was used to provide a modulated current, which was controlled by an analog square wave input from an Agilent waveform generator (33521B). Steering amplitude was measured by analysing peak separation from far-field scans performed at each modulation frequency.

### Data availability

The datasets generated during and/or analysed during the current study are available from the corresponding author on reasonable request.
